# Clinico-laboratory spectrum of dengue viral infection and risk factors associated with dengue hemorrhagic fever: a retrospective study

**DOI:** 10.1186/s12879-015-1141-3

**Published:** 2015-09-30

**Authors:** Tauqeer Hussain Mallhi, Amer Hayat Khan, Azreen Syazril Adnan, Azmi Sarriff, Yusra Habib Khan, Fauziah Jummaat

**Affiliations:** Discipline of Clinical Pharmacy, School of Pharmaceutical Sciences, University Sains Malaysia, Penang, 11800 Malaysia; Chronic Kidney Disease Resource Centre, School of Medical Sciences, Health Campus, University Sains Malaysia, Kubang Kerain 16150, Kelantan, Malaysia; Department of Obstetrics and Gynecology, School of Medical Sciences, Health Campus, University Sains Malaysia, Kubang Kerain 16150, Kelantan, Malaysia

**Keywords:** Dengue, Dengue hemorrhagic fever, Dengue shock syndrome, Risk factors, Severe dengue

## Abstract

**Background:**

The incidence of dengue is rising steadily in Malaysia since the first major outbreak in 1973. Despite aggressive measures taken by the relevant authorities, Malaysia is still facing worsening dengue crisis over the past few years. There is an urgent need to evaluate dengue cases for better understanding of clinic-laboratory spectrum in order to combat this disease.

**Methods:**

A retrospective analysis of dengue patients admitted to a tertiary care teaching hospital during the period of six years (2008 – 2013) was performed. Patient’s demographics, clinical and laboratory findings were recorded via structured data collection form. Patients were categorized into dengue fever (DF) and dengue hemorrhagic fever (DHF). Appropriate statistical methods were used to compare these two groups in order to determine difference in clinico-laboratory characteristics and to identify independent risk factors of DHF.

**Results:**

A total 667 dengue patients (30.69 ± 16.13 years; Male: 56.7 %) were reviewed. Typical manifestations of dengue like fever, myalgia, arthralgia, headache, vomiting, abdominal pain and skin rash were observed in more than 40 % patients. DHF was observed in 79 (11.8 %) cases. Skin rash, dehydration, shortness of breath, pleural effusion and thick gall bladder were more significantly (*P* < 0.05) associated with DHF than DF. Multivariate regression analysis demonstrated presence of age > 40 years (OR: 4.1, *P* < 0.001), secondary infection (OR: 2.7, *P* = 0.042), diabetes mellitus (OR: 2.8, *P* = 0.041), lethargy (OR: 3.1, *P* = 0.005), thick gallbladder (OR: 1.7, *P* = 0.029) and delayed hospitalization (OR: 2.3, *P* = 0.037) as independent predictors of DHF. Overall mortality was 1.2 % in our study.

**Conclusions:**

Current study demonstrated that DF and DHF present significantly different clinico-laboratory profile. Older age, secondary infection, diabetes mellitus, lethargy, thick gallbladder and delayed hospitalization significantly predict DHF. Prior knowledge of expected clinical profile and predictors of DHF/DSS development would provide information to identify individuals at higher risk and on the other hand, give sufficient time to clinicians for reducing dengue related morbidity and mortality.

## Background

**Fig. 1:**
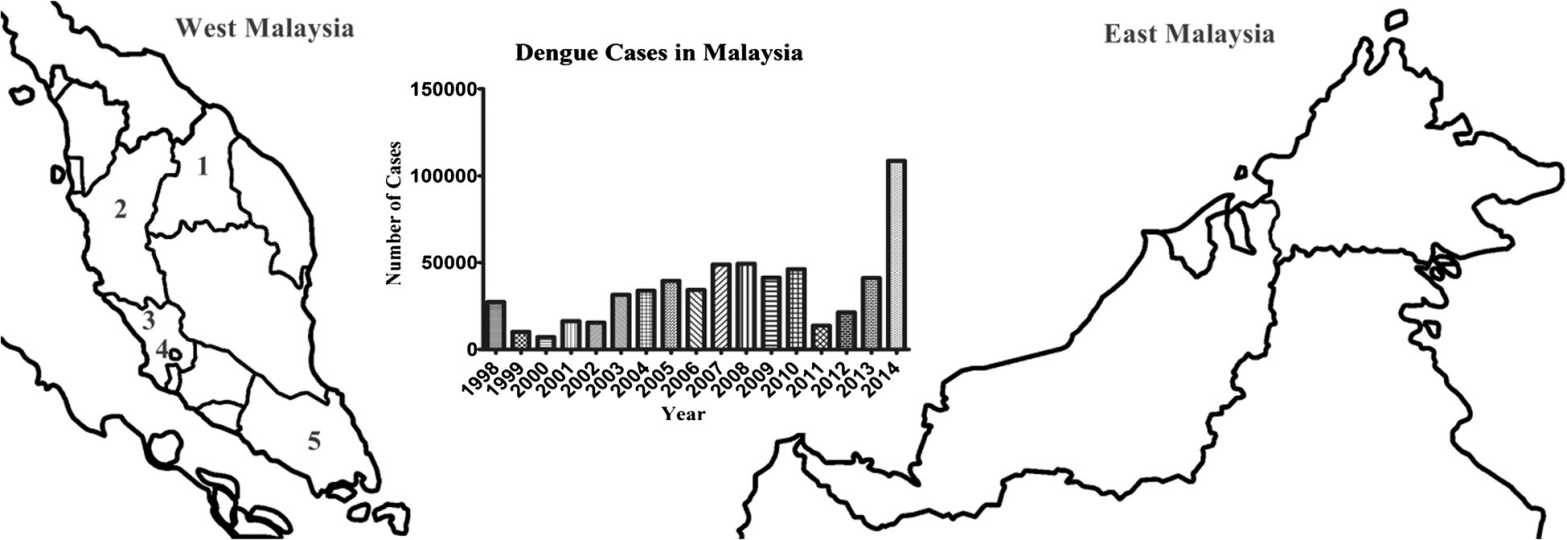
Map of top five dengue hotspots in Malaysia in 2014 (1-Kelantan, 2-Perak, 3-Selangor, 4-Kuala Lumpur, 5-Johor) and Trend of Increasing cases of dengue in Malaysia from 1998–2014

Dengue viral infection (DVI) is a dangerous and debilitating disease. Alarmingly, 40 % of the world’s population is living in the areas having a risk of being infected. WHO estimates 50–100 million dengue cases with approximately 22,000 deaths each year [1]. DVI has been an important public health concern in Malaysia ever since its first reported case in 1902 [2]. According to ministry of health Malaysia, over the past few years there is an increasing incidence of DVI with maximum number of cases observed in 2014 with a mortality rate of 0.2 % (Fig. 1). Sudden drop of dengue cases in 2011 (Fig. 1) might be attributed to the methodology difference in case reporting during this year [3].

The simultaneous presence of all four serotypes of DVI in Malaysia makes this country ’hyperendemic” for dengue [4]. Hot rainy weather, population growth, rapid urbanization, rural–urban migration, inadequacies in urban infrastructure including solid waste disposal, mega-constructions, and rise in domestic and international travel are pivotal contributing factors for drastic increase in dengue incidence in Malaysia [5].

Though mortality rate in dengue infection is not so high (<1 % with adequate medical treatment) [1], but costs associated with lost productivity and financial burden of health services have large impact on economies and households. Suaya et al, estimated that the annual cost of dengue illness (cost ± standard error) in Malaysia is 42.2 ± 4.3 million US dollars. Infection with dengue virus (DENV) appears to be a realistic threat to travellers to Southeast Asia [6]. Increasing incidence of dengue may forbid tourists to visit Malaysia that may lead to economic crisis.

Several studies have demonstrated clinico-laboratory spectrum of dengue in different regions [7–12]. Despite of drastic increase in incidence of DVI in Malaysia, there is still paucity of data to understand its clinico-laboratory spectrum. Few studies have been conducted in Malaysia to explain clinical profile of dengue infection. These studies either have concise information [13, 14], small sample size [15, 16] or included only specific population i.e. DHF, fatal cases [17] and children [18]. Therefore there is an urgent need to evaluate dengue cases more comprehensively with larger patient’s pool. For this purpose, we conducted a retrospective study in tertiary care hospital in Kelantan, Malaysia (Fig. 1).

## Methods

### Ethics statement

Study was approved by Human Resource Ethics Committee (JEPeM) of HUSM (USM/JEPeM/14080278). All data was analyzed anonymously and hence, informed consent was not required. The patients were identified from a central computerized record with their registration number (RN). Data of the cases were retrieved and specific numeral codes were given to each case before data analysis.

### Study location and participants

Current study was conducted in Hospital University Sains Malaysia (HUSM), tertiary level teaching hospital with 950 beds that serves an estimated 1.4 to 1.8 million inhabitants of Kelantan. Kelantan is an agrarian state located in the north-east of Peninsular Malaysia and among top five dengue hotspots in the country. Malays are major (95 %) ethnic group in Kelantan while Chinese constitutes merely 4 % of state population. Kelantan is one of the major hotspots of DVI where dengue cases till August 2014 (5367 case) have increased about 575 % as compared to correspondent period in 2013 (575 cases) [19]. Drastic increase in dengue cases admitted to HUSM during 2008–2014 were observed as compared to 2001–2007 (1308 vs 2123, respectively). The hospital also serves as referral centers for nearby states. Patients without confirmed diagnosis, age >12 years, concurrent co-infections (i.e. influenza, leptospirosis, malaria, typhus, yellow fever, meningitis, viral hepatitis, rickettsia, rocky mountain spotted fever and arenavirus infections) and incomplete data were excluded from the study. All suspected dengue cases admitted during Jan 2008 to Dec 2013 were taken into study but only confirmed cases fulfilling inclusion criteria were subjected to analysis.

### Dengue diagnosis, classification and laboratory tests

**Fig. 2:**
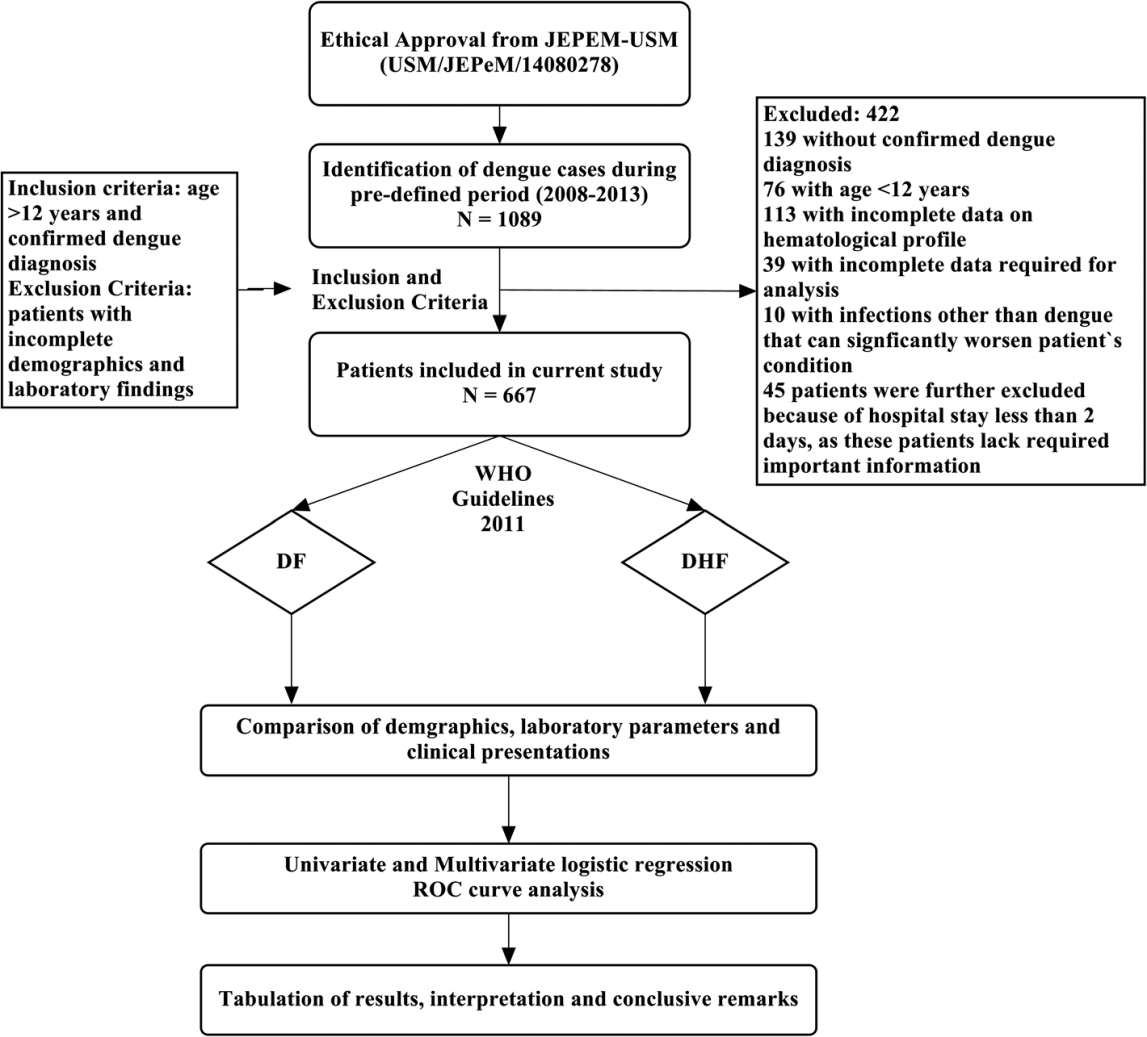
Study flow diagram

Suspected dengue infection was defined as the presence of fever and any two of the following symptoms: myalgia, headache, arthralgia, skin rash, retro-orbital pain, hemorrhagic manifestation (s), or leucopenia (white blood cell [WBC] count of <4 × 10^9^ L − 1) [20]. Suspected cases were confirmed by using at least one of the following criteria: (1) positive reverse transcriptase polymerase chain reaction (RT-PCR) result, (2) presence of dengue immunoglobulin M and G antibodies in acute phase serum by enzyme linked immunosorbent assay [Pan Bio Dengue IgM ELISA, Dengue IgM Dot Enzyme Immunoassay, SD Dengue IgM and IgG capture ELISA Kits; Standard Diagnostics, Korea], and (3) at least 4-fold increase of dengue-specific hemagglutination inhibition titers in convalescent serum when compared with acute phase serum. The serum samples were also tested for dengue-specific NS1 [pan-E Early dengue ELISA kit by Panbio, Australia and Platelia dengue NS1Ag assay by Bio-Rad Laboratories, USA) [21]. Only confirmed dengue cases were included in analysis. Primary dengue infection was distinguished from secondary infection by using IgM-IgG ratio where dengue infection was defined as primary if ratio ≥ 1.8 and as secondary if < 1.8 [22] or if there was a 4-fold increase of HAI and the titers were ≤1:1280 and ≥1:2560, respectively [23]. Serologically confirmed dengue patients were subjected to clinical case definition and disease severity was classified into DF, DHF and DSS, according to the WHO criteria [20]. Patient’s demographics and clinical presentations were recorded on day of admission while laboratory findings were recorded for each day of hospitalization until discharge. Study methodology with patient’s inclusion and exclusion criteria are shown in Fig. 2.

### Statistical analysis

Analysis was performed using SPSS software version 20.0.0. For the purpose of comparison patients were divided into DF and DHF (including grade I to grade IV). Categorical variables were recorded as frequencies and percentages while continuous variables were recorded as means and standard deviations (SD) unless otherwise stated. Categorical and continuous variables were analyzed using Chi-Square or Fischer-Exact test and independent *t*-test respectively. A logistic regression model was performed to determine the factors independently associated with severe form of dengue infection (DHF). The variables with *P* values less than 0.25 in univariate were considered as candidates for multivariate analysis. The use of univariate *P* values <0.25 has advantage of tending to include more variables in multivariate analysis while traditional levels of *P* value such as 0.05 can fail in identifying variables known to be important [24]. Receiver operating characteristics (ROC) curve analysis was used to determine the area under the curve (AUC) for prediction accuracy. Descriptive values below 5 % (*p* < 0.05) were considered statistically significant.

## Results

**Fig. 3:**
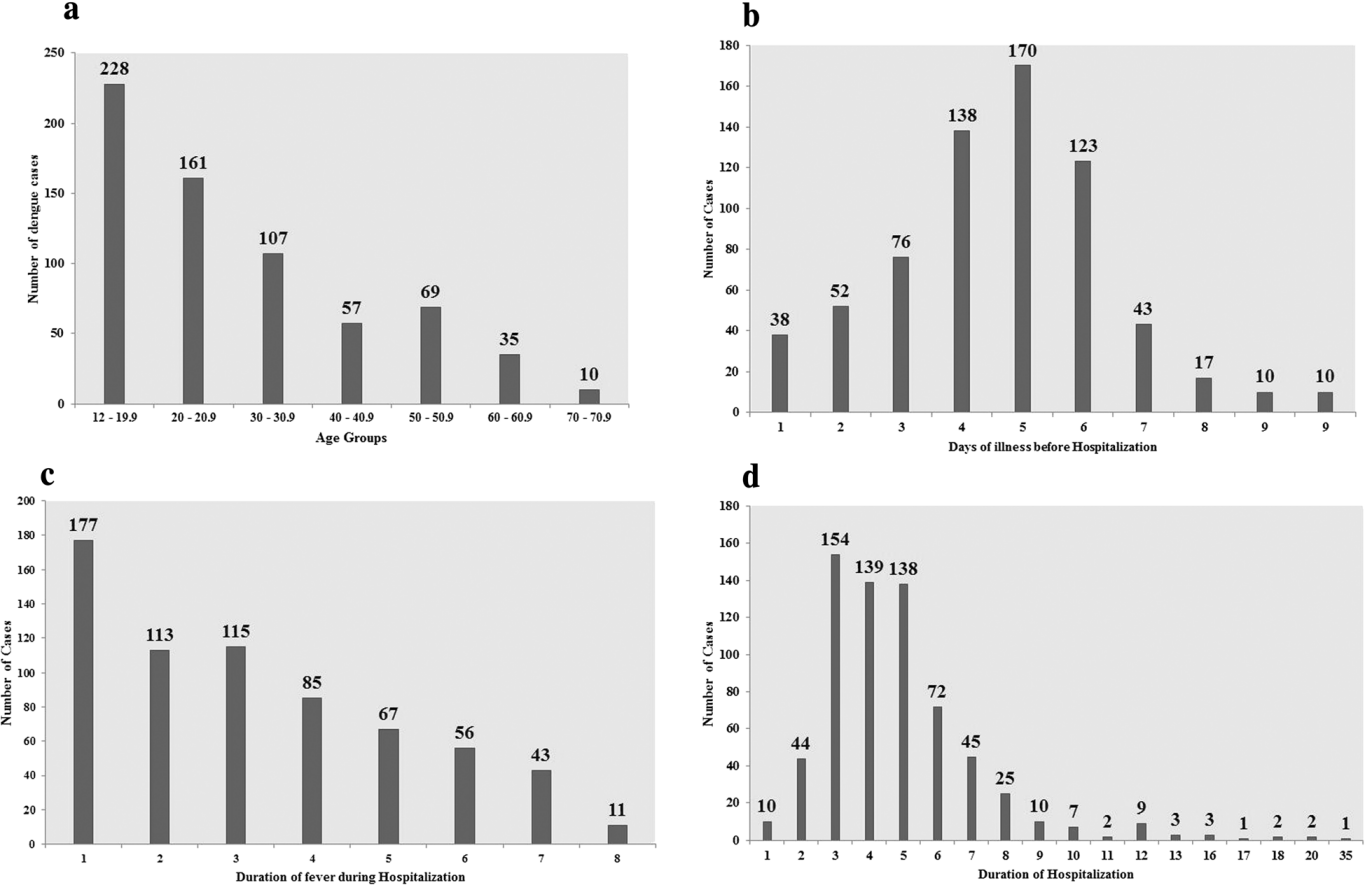
**(a)** Dengue cases in different age groups **(b)** Days of illness prior to hospitalization **(c)** Duration of fever during hospitalization and **(d)** Length of hospitalization among dengue patients

Out of total dengue cases admitted to hospital, 667 patients were included in analysis (Fig. 2). There was approximately equal distribution of gender among selected patients (male/female: 56.7 %/43.3 %, *P* = 0.062). Most of the patients (95.8 %) were adults (mean age: 30.68 ± 16.12 years) (Fig. 3a) with majority residing in urban settings (60.4 %). Ethnic Malays were predominant with 90.6 % of total cases followed by Chinese (7.6), Indians (1.5 %) and Thais (0.3 %).

Based on WHO criteria [20], DF was observed in 88.2 % (588/667) while DHF (grade I and II) and DSS (DHF grade III & IV) were observed in 11.1 % (69/667) and 0.7 % (10/667) cases, respectively. None of the patients with DF and DHF progressed to severe disease i.e. DHF and DSS respectively. Dengue infection risk groups - including family history of dengue, living in non-fogging zone, near stagnant water resources or near construction sites and travelling to jungle or to areas having high epidemics of dengue infection - were assessed in all patients. Association of patients with risk groups was observed in 40 % patients. Family history of dengue was observed in 34 % cases while 23.5 % and 18.6 % patients were living near stagnant water resources and constructions sites, respectively. Twenty seven (4 %) cases were lived in areas where fogging was not done prior to one month of their admission.

Tourniquet test (HESS test) was performed in 149 patients (positive: 101, negative: 48). Most of the patients were presented with typical dengue complaints i.e. fever, myalgia, arthralgia and headache (Table 1). Skin rash (56.9 %), lethargy (39.2 %), rigors (35.4 %), dehydration (25.3 %), shortness of breath (17.7 %), pleural effusion (10.1 %), thick gall bladder (5.1 %) and hemorrhagic anomalies (gingival bleeding, epistaxis, and hematemesis) showed a high correspondence with DHF (*P* < 0.001) in our study.

**Table 1:** Clinical manifestations of dengue cases at presentation to hospital

	Overall Cases (*N* = 667) n (%)	DF (*N* = 588) n (%)	DHF (*N* = 79) n (%)	*P*–value
Commonly occurred (among >40 % population)				
Fever	647 (97)	573 (94.7)	74 (93.7)	0.076*
Myalgia	483 (72.4)	419 (71.3)	64 (61)	0.069*
Arthralgia	389 (58.3)	336 (57.1)	53 (67)	0.092
Headache	385 (57.7)	344 (58.5)	41 (51.9)	0.265
Vomiting	368 (55.2)	318 (54.1)	50 (63.3)	0.122
Abdominal pain	299 (44.8)	264 (44.9)	35 (44.3)	0.921
Skin rash	294 (44.1)	249 (41.8)	45 (56.9)	0.014
Less Common (among 11 – 39 % population)				
Diarrhea	225 (33.7)	194 (33)	31 (39.2)	0.270
Chills	224 (33.6)	199 (33.8)	25 (31.6)	0.698
Nausea	208 (31.2)	182 (31)	26 (32.9)	0.724
Anorexia	186 (27.9)	157 (26.7)	21 (26.6)	0.982
Lethargy	186 (27.9)	142 (24.1)	31 (39.2)	0.032
Retro-orbital pain	178 (26.7)	155 (26.4)	23 (29.1)	0.603
Rigors	172 (25.8)	144 (24.5)	28 (35.4)	0.037
Flushing	135 (20.2)	119 (20.2)	16 (20.3)	0.998
Cough	118 (17.7)	103 (17.5)	15 (19)	0.748
Restlessness	116 (17.4)	105 (17.9)	11 (13.,9)	0.387
Dizziness	110 (16.5)	99 (16.8)	11 (13.9)	0.512
Jaundice	87 (13)	74 (12.6)	13 (16.5)	0.337
Dehydration	78 (11.7)	58 (9.9)	20 (25.3)	0.007
Shortness of breath	73 (10.9)	59 (10)	14 (17.7)	0.040
Sore throat	76 (11.4)	64 (10.9)	12 (15.2)	0.258
Rare (among < 11 % population)				
Malaise	52 (7.8)	45 (7.7)	7 (8.9)	0.707
Dysuria	29 (4.3)	22 (3.7)	7 (8.9)	0.069*
Hepatomegaly	39 (4.3)	25 (4.3)	4 (5.1)	0.740
Confusion	33 (4.9)	27 (4.6)	6 (7.6)	0.264*
Conjunctivitis	24 (3.6)	21 (3.6)	3 (3.8)	0.756
Chest pain	24 (3.6)	22 (3.7)	2 (2.5)	>0.90*
Pleural effusion	23 (3.4)	15 (2.6)	8 (10.1)	0.003*
Ascites	15 (2.3)	15 (2.6)	4 (5.1)	0.266
Palpitation	14 (2.1)	14 (2.4)	-	-
Edema	14 (2.1)	12 (2)	2 (2.5)	0.677
Tachypnea	12 (1.8)	11 (1.9)	1 (1.3)	>0.90
Splenomegaly	9 (1.3)	6 (1)	3 (3.8)	0.079
Thick gall bladder	9 (1.3)	5 (0.9)	4 (5.1)	0.014
Anasarca	8 (1.2)	6 (1)	2 (2.5)	0.243
Asthenia	8(1.2)	7 (1.2)	1 (1.3)	>0.90
Convulsion	8 (1.2)	8 (1.4)	0	-
Tachycardia	(0.6) 4	3 (0.5)	1 (1.3)	>0.90
Hemorrhagic Manifestations				
Petechia	80 (12)	68 (11.6)	12 (15.2)	0.352
Gingival bleeding	67 (10)	48 (8.2)	19 (24.1)	< .001
Purpura/Ecchymosis	53 (7.9)	46 (7.8)	7 (8.9)	0.749
Epistaxis	35 (5.2)	19 (3.2)	16 (20.3)	< .001*
Vaginal bleeding	24 (3.6)	21 (3.6)	3 (3.8)	0.756
Hematuria	18 (2.7)	17 (2.9)	1 (1.3)	0.711
Hematemesis	11 (1.6)	7 (1.2)	4 (5.1)	< .001
Malena	4 (0.6)	0	4 (5.1)	-
Blood in stool	4 (0.6)	3 (0.5)	1 (1.3)	0.397
Hemoptysis	2 (0.3)	2 (0.3)	0	-

*Fisher’s exact test (if more than 20 % of cells with expected counts of less than 5) while all the other *P* values were calculated by Pearson Chi-Square

The mean value of serum creatinine (128.94 ± 81.20 μmol/L, *P* = 0.001), hematocrit (49.57 ± 6.22 %, *P* < 0.001), aPTT (54.13 ± 7.81 s, *P* < 0.001) and PT (13.84 ± 0.96 s, *P* < 0.001) were significantly higher among DHF cases (Table 2).

**Table 2:** Comparison of laboratory characteristics (on admission) of DF and DHF

	Overall Cases (*N* = 667) Mean ± SD	DF (*N* = 588) Mean ± SD	DHF (*N* = 79) Mean ± SD	*P*–value*
Age (Years)	30.69 ± 16.13	29.86 ± 15.67	36.80 ± 18.14	0.002
Temperature (°C)	37.68 ± 0.62	37.66 ± 0.61	37.78 ± 0.62	0.109
Pulse rate (BPM)	83.25 ± 17.57	83.30 ± 17.47	82.82 ± 18.36	0.820
Serum Creatinine (μmol/L)	99.13 ± 58.57	95.12 ± 53.58	128.94 ± 81.20	0.001
Total protein (g/L)	66.08 ± 12.02	66.40 ± 11.65	63.90 ± 14.180	0.157
Albumin (g/L)	40.26 ± 15.36	40.50 ± 16.29	38.61 ± 5.33	0.337
Globulin (g/L)	28.39 ± 5.35	28.48 ± 5.32	27.81 ± 5.51	0.318
AG ratio	1.79 ± 4.46	1.72 ± 3.91	2.29 ± 7.28	0.510
AST (IU/L)	152.42 ± 209.76	135.14 ± 184.53	185.03 ± 241.30	0.034
ALT (IU/L)	114.36 ± 166.64	113.31 ± 161.38	121.54 ± 200.13	0.743
ALP (IU/L)	105.85 ± 71.33	106.27 ± 70.99	102.92 ± 74.02	0.711
Total bilirubin (μmol/L)	12.29 ± 18.40	12.36 ± 19.32	11.77 ± 10.39	0.669
WBCs (cells × 10^9^/L)	5.16 ± 14.78	5.29 ± 15.73	4.17 ± 2.75	0.531
Thrombocytes (cells × 10^9^/L)	97.59 ± 63.15	99.63 ± 65.56	82.74 ± 38.75	0.038
Hemoglobin (g/dL)	14.21 ± 7.98	14.18 ± 8.48	14.48 ± 1.52	0.750
Hematocrit (%)	41.10 ± 6.23	39.92 ± 5.25	49.57 ± 6.22	0.000
INR	1.06 ± 0.56	1.06 ± 0.60	1.04 ± 0.10	0.821
APTT (Sec)	43.62 ± 10.47	41.96 ± 9.86	54.13 ± 7.81	0.000
PT (Sec)	12.90 ± 1.37	12.84 ± 1.38	13.84 ± 0.96	0.000
Period of illness prior to hospitalization	4.16 ± 3.28	2.89 ± 1.84	4.45 ± 1.60	0.033
Duration of fever during hospitalization	4.21 ± 2.11	3.21 ± 2.60	4.60 ± 3.47	0.006
Length of Hospitalization (days)	4.88 ± 2.74	2.52 ± 1.92	4.64 ± 1.99	0.026

*Student *t* test or Mann–Whitney *U* test, where appropriate

Longer duration of hospitalization and prolonged fever was observed in patients with DHF. Similarly, patients with DHF admitted late to hospital than patients with DF. We found that patients having age >40 years, urban residency, secondary infection and warning signs were more likely to have severe form of dengue infection (DHF/DSS) (Table 3).

**Table 3:** Comparison of demographics and clinical features of DF and DHF

	Overall Cases (*N* = 667) n (%)	DF (*N* = 588) n (%)	DHF (*N* = 79) n (%)	*P*–value*
Age > 40 years	167 (25)	137 (23.3)	30 (38)	0.005
Male gender	378 (56.7)	326 (55.4)	52 (55.8)	0.08
Urban resident	403 (60.4)	346 (41.2)	57 (72.2)	0.023
Temperature > 38 C^o^	229 (34.3)	197 (33.5)	32 (40.5)	0.218
Obesity	94 (14.1)	82 (13.2)	12 (15.2)	0.467
Co-morbidities				
DM	36 (5.4)	13 (2.2)	23 (29.1)	0.001
HTN	35 (5.2)	17 (2.9)	18 (22.4)	0.021
CKD	33 (4.9)	26 (4.4)	7 (8.9)	0.085
IHD	25 (3.7)	19 (3.2)	6 (7.6)	0.178
CHF	2 (0.3)	2 (0.3)	0 (0)	-
HPL	8 (1.2)	7 (1.2)	1 (1.3)	0.649
Risk group association^a^	264 (39.6)	232 (39.5)	32 (40.5)	0.858
Secondary infection	73 (10.9)	58 (9.9)	18 (22.8)	0.015
Warning signs	271 (40.6)	224 (38.1)	47 (59.7)	0.000
Positive Hess test	101 (15.1)	85 (14.5)	16 (20.3)	0.177
Low MAP	37 (5.5)	34 (5.8)	3 (3.8)	0.469
Elevate Scr (AKI)	95 (14.2)	58 (9.9)	37 (65.8)	0.000
Thrombocytopenia	395 (59.2)	343 (58.3)	56 (70.8)	0.042
Leukopenia	363 (54.4)	321 (54.6)	42 (53.2)	0.596
Leukocytosis	36 (5.4)	32 (5.4)	4 (5.1)	0.842
Elevated ALT	362 (54.3)	315 (53.6)	47 (59.5)	0.321
Elevated AST	447 (67)	380 (64.6)	67 (84.8)	0.000
Elevated ALP	113 (19.9)	117 (19.9)	16 (20.3)	0.885
Transaminitis	360 (54)	311 (52.9)	49 (62)	0.125
Rhabdomyolosis	49 (7.3)	34 (5.8)	15 (19)	0.000
Multiple organ dysfunctions	138 (20.7)	97 (16.5)	43 (54.4)	0.000
Urinary sedimentations	96 (14.4)	78 (13.3)	18 (22.8)	0.034
Hemoconcentration	73 (10.9)	56 (9.5)	17 (21.5)	0.001
Prolonged PT and aPTT	116 (17.4)	93 (15.6)	23 (29.1)	0.003
Delayed Hospitalization (>3 days of onset of illness)	153 (22.9)	105 (17.9 %)	48 (60.8)	0.000
Death	8 (1.2)	6 (1)	2 (2.5)	0.243

*Chi-Square/Fisher’s Exact test, where appropriate, ^a^defined as number of patients having association of dengue infection risk groups as described under results section

**Fig. 4:**
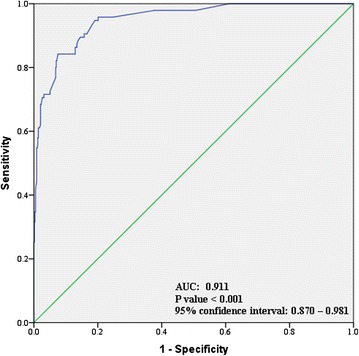
ROC Curve analysis of logistic regression model to predict DHF

To identify possible risk factors of DHF among dengue patients, logistic regression analysis was performed for clinically relevant and statistically tested variables. Out of five tested signs/symptoms, lethargy (OR: 3.1, *P* = 0.005) and thick gallbladder (OR: 1.7, *P* = 0.029) were two symptoms with a higher likelihood of presenting DHF. Patients with age greater than 40 years, secondary infection and diabetes mellitus presented a higher risk of DHF in our study (Table 4). It was also observed that patients who were admitted after 3 days of onset of illness (delayed hospitalization), were associated with a higher risk (OR: 2.3, *P* = 0.037) of having DHF than patients who were admitted within three days (Fig. 3b). ROC curve analysis of logistic model is shown in Fig. 4.

**Table 4:** Univariate and Multivariate logistic regression analysis for risk factors of DHF

Variables	Univariate analysis			Multivariate analysis		
	*P*-value	OR	95 % CI	*P*-value	OR	95 % CI
Age > 40 years	0.005	2.0	1.23 – 3.31	<0.001	4.1	2.12 – 5.71
Secondary Infection	0.017	2.1	1.15 – 3.90	0.042	2.7	1.64 – 3.91
Diabetes mellitus	0.020	3.9	1.34 – 4.21	0.041	2.8	1.35 – 4.67
Hypertension	0.047	3.7	1.33 – 3.11	0.211	2.3	2.45 – 7.45
Skin rash	0.150	1.8	1.12 – 2.90	0.346	2.6	1.88 – 4.78
Lethargy	0.023	2.2	0.81 – 3.10	0.005	3.1	1.89 – 5.11
Retro-orbital Pain	0.604	1.2	0.68 – 1.93	-	-	-
Rigors	0.061	1.7	1.03 – 2.79	0.062	2.2	0.98 – 3.78
Thick gallbladder	0.007	6.2	1.63 – 23.67	0.029	1.7	0.78 – 2.12
Delayed hospitalization	0.008	2.7	1.88 – 4.11	0.037	2.3	1.34 – 2.89

Variables with *P* < 0.25 (retro-orbital pain) were excluded from multivariate analysis

Odds ratio (OR) and Confidence interval (CI) have been rounded off

Eight patients (1.2 %) died during study period (DF: 6, DHF: 2). Age > 40 years, secondary infection and warning signs were observed in 6 (75 %), 3 (37.5 %), and 5 (62.5 %) fatal cases, respectively. All of the fatal cases in our study were admitted on day 5 of onset of illness and were accompanied by MODs. Shock (2/6), respiratory failure (2/2) and renal complications (4/4) were primary causes of death. Abnormal renal and hepatic anomalies were observed in 12.1 % and 35.5 % of studied participants at discharge (Table 5).

**Table 5:** Renal and Hepatic anomalies among dengue patients at discharge

	DF (*N* = 588) n (%)	DHF (*N* = 79) n (%)
Normal renal functions	522 (88.8)	64 (81)
Renal insufficiencies (SCr <200 μmol/L)	61 (10.4)	14 (17.7)
Renal insufficiency (SCr > 200 μmol/L)	5 (0.9)	1 (1.3)
Normal hepatic function	382 (65)	48 (60.8)
Mild to moderate transaminitis (2–10 × ULN)	175 (29.8)	27 (34.2)
Severe transaminitis (>10 × ULN)	31 (5.3)	4 (5)

### Study limitations

Being a retrospective study, some limitations needed to be addressed. All the reported values are dependent on the thoroughness of clinician’s documentation. Clinical outcomes of patients may be biased due to lack of standardized dengue management protocol, and the use of different management strategies to treat DVI. Viral load was not assessed in current study. Furthermore, patients were not followed up to assess full recovery. Risk factors of mortality were not assessed because of few fatal cases that negatively influence statistical power of current study. However, the strength of the current study is comprehensiveness, inclusion of patients during dengue peak season (rainy season) and outbreaks.

## Discussion

Current study is first comprehensive evaluation of DVI in Kelantan, one of the major dengue hotspots in Malaysia. Recent surge of DVI in Malaysia is the result of change in variation of dengue virus (DENV). All four serotypes of DENV are prevalent in Malaysia. Alarmingly, discovery of fifth serotype (DENV-5) in Malaysia demands more authoritative measures in terms of surveillance, prevention and treatment [25]. We studied DVI cases to understand clinico-laboratory characteristics among multiethnic population of Malaysia.

Initially dengue infection was thought to be a disease of children but recently it has been reported that age distribution of this disease has shifted to adults and older age [26, 27]. Increase mobility of adult population in our society, better access to health care facilities and ease of reporting to physicians might be some causative factors of high incidence of DVI among adults. Similar trend was observed in our study where prevalence of dengue infection was higher among patients having age 20–40 years than patients with age <20 years (Fig. 4a). In Malaysia, incidence of dengue among pediatric population is declining while incidence among adult population has been on the risk, as in 2006 about 80 % reported cases to ministry of health had age > 15 years [28]. The trend for increased incidence among adults has important implications for control and prevention. On the other hand, increasing age was also associated with DHF in our study where patients with age > 40 years were associated with four times odds of having DHF (Table 4). It might be due to presence of secondary infection that is believed to increase the risk of more serious disease. In endemic areas, adults and older children are likely to have past exposure to dengue infection and also an increasing risk for secondary infection and thus severe infection [22]. Out of 30 DHF patients with age > 40 years, secondary infection was present in approximately half of the cases. Additionally, generalized decrease in immunity due to modified cellular and humoral immune responses with increasing age might be some other contributing factors of severe disease in advance ages [29]. Furthermore, impact of age on clinico-laboratory spectrum of disease has already been reported [30]. In our study the patients with age 12–18 years had higher prevalence of cough, abdominal pain, skin rash, gum bleeding, high fever, plasma leakage, higher respiratory and heart rate as compared to patients with age >18 years. These findings are consistent with previous studies [30, 31] and suggest presence of varying clinical manifestations of dengue in different age groups. Higher number of urban residents (60.4 %) in our study might be due to location of hospital surrounded by urban areas. Additionally, urbanization also favors vector breeding. Males were found to be affected by DVI slightly more than females in our cohort, but this difference was not statistically significant (*P* = 0.062). These findings are consistent with results of Anker & Arima [32]. In contrast, recently higher prevalence of DVI in female has also been observed [33].

Since patients with mild or classical DF can develop severe infection later, therefore it is important to look for sings/symptoms to facilitate the early prediction of severe dengue i.e. DHF/DSS. The clinical manifestations might always offer the earliest marker in predicting severe disease. Therefore dengue with warning signs should be monitored vigilantly in order to avoid its progression to severe disease [34]. Presence of warning signs was significantly associated with DHF in our study (Table 3). According to logistic regression analysis, thick gallbladder (OR: 1.7) and lethargy (OR: 3.1) were more likely to be associated with DHF, while skin rash, retro-orbital pain and rigors were not significant risk factors (Table 4). Furthermore, dehydration, dyspnea, pleural effusion, gingival bleeding, epistaxis and hematemesis were more profound clinical presentations among DHF patients (Table 1). These clinical presentations and warning signs were not included in multivariate analysis because they are significantly relevant with DHF and were serve as diagnostic tool in clinical case definition of dengue [20]. Similarly, presence of certain co-morbidities like diabetes mellitus, hypertension, chronic kidney disease, allergies, asthma, ischemic heart disease and hepatic anomalies might place some patients at high risk of developing DHF/DSS. We found statistical association of diabetes mellitus (DM) with DHF, where individuals suffering from DM had higher odds of developing DHF than patients without disease. Hypertension was more profound (*P* = 0.021) among DHF cases (Table 3) but did not show any statistical association with development of DHF, though unadjusted estimates suggested that patients who had hypertension were 1.7 times at higher risks of developing DHF (Table 4). These findings are persistent with previous literature [35, 36]. Increased capillary fragility and permeability due to activation of T-lymphocytes and release of cytokines in DM are might be some possible factors of development of DHF [35, 36].

Approximately, 40 % patients in our study had risk groups association (Table 3). These risk groups were family history of dengue infection and living near non-fogging zones, near stagnant water resources or construction sites. These findings will aid health authorities to initiate appropriate vector control measures in affected areas. Most of the dengue cases in our study occurred during months of mid-November to end of December. Heavy rain fall during these months might be a contributing factor for increased incidence of DVI. Preventive measures with full swing should be carried out before these months in order to combat this disease.

Fever among dengue patients typically lasts for 2–7 days. Total duration of fever in our study ranges from 1–8 days (Fig. 4c), among them prolonged fever was more profound among DHF cases (mean 4.9 days, *P* < 0.006). Longer duration of fever among dengue patients ranges from 10–16 days has also been observed [8]. All the other possible causes of fever were ruled out and no other cause was found. Fever was resolved within 4 days of admission in most of the patients. Prolonged fever was found to be associated with longer hospital stay in our study. Average duration of hospital stay was 4.88 days and DHF was associated with significantly longer hospital stay (Table 2, Fig. 4d) resulting in significant burden in terms of cost of care. This is of particular importance in resource limited setting, especially in dengue endemic regions. Similarly, DF required hospital stay >3 days in our study indicating that both DF and DHF impose a considerable burden in the health care system.

Unusual manifestations of patients with severe organ involvement such as liver, kidneys, brain or heart associated with dengue infection have been increasingly reported in DHF and also in dengue patients who do not have evidence of plasma leakage. In recent dengue classification, these manifestations are termed as “expanded dengue syndrome” [20]. Liver was most affected organ in our study and deranged hepatic enzymes were present in most of the patients. Grossly elevated liver enzymes are known to be associated with DHF and major bleeding [16]. Mean levels of AST were significantly differing between DF and DHF in our study (Table 2). Elevated ALT and ALP was also observed in DHF patients, but difference was not statistically significant (*P* > 0.05). Additionally, hepatomegaly accounted 5.1 % and 4.3 % among DHF and DF patients, respectively (Table 1). Dengue virus (DENV) directly affects hepatocytes (Kupffer cells) resulting in elevated liver transaminases and hepatomegaly. Hepatocellular damage in dengue infection is might be attributed to activated T – lymphocyte subsets, being more evident in DHF than in DF [16]. Wahid et al. [16] demonstrated that extent of hepatocellular damage can be predicted by spontaneous bleeding, that was found in 9.7 % cases in our study. In addition, hypoalbuminemia was more profound among patients with DHF than with DF, though difference was not statistically significant (Table 2). These results are consistent with previous findings where hypoalbuminemia is often present in dengue infection [37, 38]. Our results suggest that liver impairments are more common in DHF compared to DF.

Kidney was second most affected organ after liver in our study. Acute kidney injury (AKI) defined by AKIN criteria was present in 14.2 % of total population and was more commonly associated with DHF (Table 3). Similarly, urinary sedimentations were found in 14.4 % cases, more profoundly among DHF cases (Table 3). Several reports have described that patients with DHF/DSS are more likely to have AKI (Mallhi et al.) [39]. Spectrum of dengue induced nephropathies ranges from proteinuria to severe AKI and can be explained by the direct viral injury or antigen-antibody complex in glomeruli [40].

Dengue virus has broader tropism and can not only replicate in hepatocytes and glomeruli but also in type II pneumocytes, cardiac fibers, as well as in resident and circulating monocytes/macrophages and endothelial cells leading to multiple organ dysfunctions (MODs) [41]. MODs can be considered as a sequential or concomitant occurrence of a significant derangement of function in two or more organ systems of the body, against a background of a critical illness [42]. We used same criteria in our study and found significantly higher prevalence of MODs (*P* < 0.001) among DHF cases than DF (54.4 % vs 16.5 %). Acute pancreatitis, type 1 respiratory failure, circulatory failure and rhabdomyolosis were observed in 6 (1 %), 11 (1.6 %), 3 (0.4 %) and 49 (7.3 %) patients respectively. MODs caused by bleeds into various organs are also associated with higher mortality among DHF patients [42]. In current study, MODs were observed in all eight fatal cases where AKI was observed in 8, liver failure in 4, pancreatitis in 4, respiratory failure in 2 and circulatory failure in 2 patients.

Hematological profile of dengue patients is usually served to differentiate DF and DHF [43]. Hematocrit has been used as an important parameter in monitoring patients with DF. According to Malaysian clinical practice guidelines [44], hematocrit values of 47 % for male and 40 % for female were suggested for cut-off value to suspect plasma leakage. These values were validated in our study and we found hemoconcentration, characterized by 20 % raised hematocrit, was significantly associated with DHF (Table 3). Similarly, thrombocytopenia was more common among DHF patients and these findings are consistent with previous reports [8, 13]. Hemoconcentration in our study might be due to dehydration and increased vascular permeability [22]. However, reduction of hematocrit levels to normal was more significant in DHF than DF and it may be influenced by vigorous intravenous therapy in DHF in current study. Besides these, simultaneous elevation of both PT and aPTT was more significant among DHF (Table 3) and it can be attributed to disturbance in balance of coagulation cascade pathways (prothrombotic, antithrombotic and fibrinolytic pathways). Low levels of circulating protein C, S, antithrombin III and elevated levels of tissue factor, thrombomodulin, PAI-1 are likely to be related to hemorrhage, plasma leakage and shock in dengue infection [45].

Diagnostic delays may complicate clinical state of the dengue patients [46]. On average the patients in current study were admitted on day four of illness (Table 2) and delayed hospitalization was found to be more common among patients with DHF. These findings are consistent with a Mexican study where diagnostic delay >2 days was significantly associated with hemorrhagic cases [46]. A study in Cuba also reported that hospitalization of patients at an average of 2.9 days was associated with worsening clinical condition [47]. Late hospitalization may also be a possible contributing factor of rapid deterioration in severe dengue and our data clearly demonstrated that patients with delayed hospitalization had 2.3 times higher risks of developing DHF than those who hospitalized within three days of onset of illness (Table 4).

Dengue viral infections are rarely fatal, although fatal infections do occur [38] due to plasma leakage, fluid accumulation, respiratory distress, severe bleeding or multiple organ involvement [41]. Overall mortality in our study was 1.2 % and all death cases had worst clinical presentations and were accompanied by MODs in our study. These findings are consistent with study of Leo et al. [12]. Additionally, died patients were admitted on day five of illness and most of them had defervescence, followed by rapid deterioration of clinical condition. Our observation is consistent with an earlier study done on dengue deaths where late hospitalization was found to be a possible contributing factor to increased risk of mortality [17].

In current study, patients were managed according to their clinical conditions. Hydration status was maintained either by oral or intravenous routes. Patients were treated with acetaminophen and H_2_ receptors blockers/proton pump inhibitors for fever and gastrointestinal disturbances, respectively. Twenty three (3.9 %) patients with DF and 16 (23.3 %) patients with DHF received blood transfusion in our study. Most of the patients were fully recovered at discharge but hepatic and renal anomalies were present in 35.5 % and 12.1 % patients respectively (Table 5).

## Conclusions

Dengue viral infection is a dangerous and debilitating disease that is a growing threat to the global health. Malaysia is facing worse dengue crisis where death toll due to dengue have raised to danger level. Our findings showed that dengue is common in all age groups regardless of gender, race and residency. In current study, dengue presented with several typical and some atypical manifestations. Our data showed that DF and DHF presented with significantly different clinico-laboratory characteristics where DHF is fatal and highly morbid disease accompanied by multiple organ dysfunctions and longer hospital stay. Presence of age > 40 years, secondary infection, diabetes mellitus, lethargy, thick gallbladder and delayed hospitalization can predict high risk patients for developing DHF. Understanding the predictor of DHF/DSS development would provide information to identify individuals at higher risk and on the other hand, give sufficient time to clinicians for reducing dengue related morbidity and mortality. Routine use of laboratory values in diagnosis of dengue coupled with public awareness and vigilant monitoring by health care professionals could go a long way in combating dengue. Our findings will help national dengue control authorities to continue strive for prevention and treatment of highly incident and dangerous tropical disease such as DVI.

## Abbreviations

AG ratio: Albumin globulin ratio
AKI: Acute kidney injury
ALP: Alkaline phosphatase
ALT: Alanine amino transferase
ALT: Alanine aminotransferase
aPTT: activated prothrombin time
AST: Aspartate aminotransferase
BPM: Beats per minute
BUN: Blood urea nitrogen
CHF: Congestive heart failure
CKD: Chronic kidney disease
DF: Dengue fever
DHF: Dengue hemorrhagic fever
DM: Diabetes mellitus
DSS: Dengue shock syndrome
DVI: Dengue viral infection
Hct: Hematocrit
HPL: Hyperlipidemia
HTN: Hypertension
IHD: Ischemic heart disease
INR: International normalized ratio
MAP: Mean arterial pressure
PT: Prothrombin time
SCr: Serum creatinine
ULN: Upper limit normal
WBCs: White blood cells
